# DiaBD: A diabetes dataset for enhanced risk analysis and research in Bangladesh

**DOI:** 10.1016/j.dib.2025.111746

**Published:** 2025-05-31

**Authors:** Tabia Tanzin Prama, Md. Jobayer Rahman, Marzia Zaman, Farhana Sarker, Khondaker A. Mamun

**Affiliations:** a Advanced Intelligent Multidisciplinary Systems (AIMS) Lab, Institute of Research, Innovation, Incubation, and Commercialization (IRIIC), United International University, Dhaka 1212, Bangladesh; b CMED Health Ltd., Mohakhali DOHS, Dhaka, 1206, Bangladesh; c Center for Computational and Data Sciences, Independent University, Bangladesh, Dhaka 1229, Bangladesh; d Department of Computer Science and Engineering, Independent University, Bangladesh, Dhaka 1229, Bangladesh; e Department of Computer Science and Engineering, United International University, Dhaka 1212, Bangladesh

**Keywords:** Diabetes, Medical care, Biometric data standardization, Risk factor analysis, Predictive analysis

## Abstract

Diabetes is a chronic condition affecting millions worldwide and severely impacts health and quality of life. According to the International Diabetes Federation (IDF), over 463 million adults, which is 9.3% of the global population, live with diabetes. Diabetes ranks among the most prevalent chronic diseases and was the ninth-leading cause of mortality in 2019, with 4.2 million deaths reported. This article introduces DiaBD, a novel dataset of 5,288 patient records from Bangladesh, designed to address critical gaps in diabetes research and aid in healthcare planning, risk analysis, and predictive modelling. The dataset comprises 14 attributes including age, gender, clinical vitals (pulse rate, systolic and diastolic blood pressure, glucose levels), anthropometrics (height, weight, body mass index (BMI)), family history of diabetes and hypertension, cardiovascular disease (CVD), and stroke, with a dependent attribute, Diabetic, indicates whether an individual has diabetes or not. The dataset ensures demographic diversity and precise measurements, supporting the study of diabetes and its related health issues. Features like CVD and stroke enable broader research on comorbidities. This dataset facilitates machine learning applications, risk assessment, and personalized healthcare strategies. Researchers can explore the links between diabetes, hypertension, CVD, and stroke, while healthcare providers and policymakers can leverage DiaBD to identify trends, allocate resources efficiently, and enhance public health strategies.

Specifications Table

**Table:** 

Subject	Computer Sciences Health Sciences, Medical Sciences & Pharmacology
Specific subject area	Diabetes Data Analysis, Type-2 Diabetes Risk Analysis.
Type of data	Table, Raw, Analyzed, Filtered.
Data collection	Community health workers (CHWs) collected data from 5,288 participants across 63 diverse Bangladeshi Unions using a smart health kit and an AI-based app under the Enriched Sastho program. They gathered categorical and quantitative measures, including vitals like blood pressure and glucose, with tools such as a Sphygmomanometer and Glucometer. The dataset included individuals aged 21–80 years, with incomplete records excluded. Measurements followed WHO guidelines, and the data were validated to ensure the dataset highest standard.
Data source location	Institution: CMED Health Ltd. Palli Karma-Sahayak Foundation (PKSF) Country: Bangladesh
Data accessibility	Repository name: Mendeley DOI: 10.17632/m8cgwxs9s6.2 Direct URL to data: https://data.mendeley.com/datasets/m8cgwxs9s6/2 Instructions for accessing this data: The dataset is publicly available in CSV format and does not require any access controls. License: CC BY 4.0 CC – Free to use with credit. Link license, note changes, and no endorsement. Third-party content may need permission.
Related research article	None

## 1 Value of the Data

• The dataset includes key attributes such as blood pressure, glucose levels, BMI, hypertension, stroke, and CVD, which provide comprehensive information on diabetes and its complications to enable the development of effective preventive and therapeutic measures. The prevalence of diabetes within the dataset is approximately 6.5%, with 342 diabetic individuals out of 5,288 total participants.
• The dataset can be crucial for developing and validating machine learning algorithms for early diabetes detection, risk assessment, and prediction of disease progression. We have used the dataset to train machine learning (ML) models to predict diabetes, where key attributes include age, gender, pulse rate, blood pressure, hypertension, family history, glucose, BMI, height, weight, stroke, CVD, and diabetes status. Preliminary analysis using ML models, Random Forest (RF) classifier showed promising results in classifying diabetic and non-diabetic individuals. The classification algorithm achieved the highest performance with 99.26% accuracy and 0.99 AUC for DiaBD, 85.56% accuracy and 0.84 AUC for PIMA, and 91.7% accuracy and 0.92 AUC for Ranchi.
• This dataset addresses key research gaps in Low- and middle-income countries (LMICs) by providing region-specific data that is often under-represented in global studies. Unlike the PIMA dataset, which focuses on Pima Indian women, our dataset includes a diverse demographic from urban, semi-urban, and rural regions of Bangladesh, with male and female participants aged 21 to 80 years. This diversity improves the generalizability of the findings to Bangladesh and similar LMICs, where diabetes research lacks representation. Comparative studies with datasets such as PIMA highlight region-specific disparities and risk factors.
• Researchers can assess the effectiveness of diabetes treatments and analyze how demographics and medical conditions (e.g., hypertension, CVD) influence outcomes. The dataset helps design personalized medicine strategies by identifying high-risk individuals based on their health data. Healthcare professionals can use this data to identify trends, design targeted treatment plans, and improve patient care. Additionally, the dataset supports the development of predictive models for clinical decision-making.
• DiaBD can be reused in health informatics, public health, and epidemiology to study risk factors, intervention strategies, and healthcare analytics. It can be integrated with external data sources that could help analyze broader trends in diabetes and its comorbidities in different regions or populations.

## 2 Background

Diabetes mellitus (DM) is a metabolic disorder characterized by persistently high blood glucose levels that lead to symptoms like frequent urination, thirst, and hunger [1]. If untreated, DM can cause severe complications such as diabetic ketoacidosis or death [2,3] and damage blood vessels, raising risks of heart attack, stroke, kidney disease, and vision loss [4,5]. The complexity and wide-ranging impact of diabetes necessitate robust datasets for accurate research and risk assessment [6,7]. Researchers gain the most comprehensive insights by collecting data using mixed-method techniques [8]. There are some problems with existing datasets like the PIMA dataset [9], the Ronchi University dataset [10], the Luzhou, China dataset [11], and the Reza et al. dataset [12]. These problems include small sample sizes, poor demographic representation, and limited clinical or lifestyle parameters. These limitations reduce the dataset’s generalizability to broader populations and modern healthcare contexts.

The DiaBD dataset [13] aims to bridge gaps by incorporating wider array of clinical, demographic, and lifestyle variables. This feature set and a more diverse population enhance the dataset’s relevance and utility for diabetes research. The dataset offers a modern resource to assess diabetes risk factors, which is ideal for developing robust predictive models and supporting prevention and management.

## 3 Data Description

This research describes a new set of data that includes gender and age, as well as critical clinical measurements like height, weight, blood pressure, pulse rate, glucose levels, and BMI. It also includes information about hypertension, medical history, such as family history of diabetes and high blood pressure, and current conditions such as stroke and heart disease. This comprehensive dataset is a foundation for developing prediction models for early diabetes detection and improved diabetes management. The description of those attributes is outlined in Table 1.

**Table 1:** Description of dataset attributes.

Feature	Data Type	Description
Age	Integer	Patient’s age in years
Gender	Categorical	Male, Female
Pulse Rate	Float	Number of times the heart beats per minute (bpm)
Systolic BP	Float	The pressure exerted by blood against the walls of arteries during each heartbeat. Typical SBP values for adults are less than 120 mmHg.
Diastolic BP	Float	The pressure exerted by blood against the walls of arteries when the heart is at rest between beats. Typical DBP values for adults are less than 80 mmHg.
Hypertension	Boolean	Hypertension, or high blood pressure, is defined as blood pressure consistently at or above 140/90 mm Hg. (No=0 or Yes=1)
Family Hypertension	Boolean	Having one or both parents with hypertension. (No=0 or Yes=1)
Family Diabetes	Boolean	Having a parent, sibling, or other close relative with type 2 diabetes. (No=0 or Yes=1)
Glucose	Float	The blood contains the amount of glucose (sugar) at a given time. The normal blood glucose level range is typically between 3.9 and 5.5 millimoles per liter (mmol/L) when fasting and up to 7.8 mmol/L after meals.
Body Mass Index (BMI)	Float	The BMI is a measurement of a person's body fat based on their height and weight. Body mass index = (weight in kg/(height in m)^2)
Height	Float	Height (Meter)
Weight	Float	Weight (KG)
Stroke	Boolean	Affected by stroke (No=0 or Yes=1)
Cardiovascular Disease (CVD)	Boolean	Having cardiovascular disease (No=0 or Yes=1)
Diabetic	Boolean	Class variable (No=Non-diabetic or Yes=Diabetic)

**Fig. 1:**
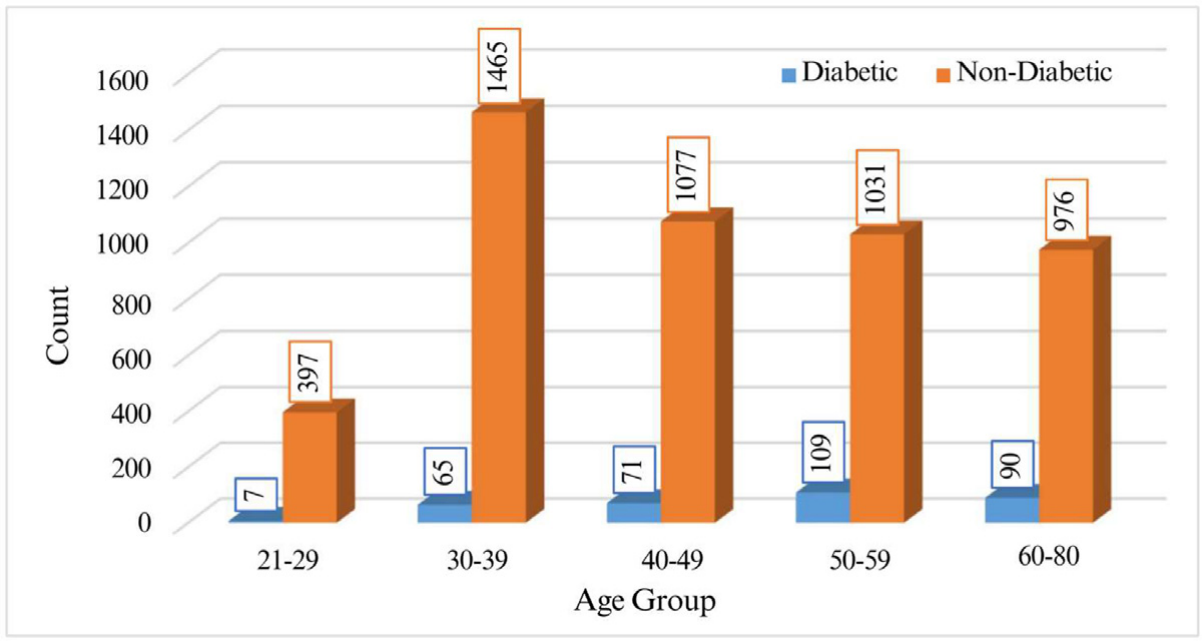
Frequency distribution of diabetic and non-diabetic participants by age group.

The dataset integrates age-related data across several generational categories, ranging from 21 to 80 years, ensuring a comprehensive demographic analysis. This broad coverage allows for an accurate assessment of diabetes occurrence across different life stages. Fig. 1 illustrates the frequency distribution of diabetic and non-diabetic individuals by age group, highlighting variations in diabetes incidence within the dataset.

Gender plays a critical role in diabetes research, as it can influence the risk factors, progression, and management of the disease. This dataset includes male and female participants, consisting of 3,752 females and 1,536 males. Such diverse demographic sampling facilitates a comprehensive analysis of diabetes prevalence across genders. Expert physicians have validated and collected the dataset's characteristics and all associated values multiple times to ensure the reliability of this research. Based on the most recent data, Fig. 4 illustrates the gender-specific distribution of diabetic and non-diabetic individuals, highlighting distinct diabetes rates between men and women. Table 2 shows the summary statistics for continuous and categorical variables. We present continuous variables, such as age, glucose levels, BMI, and blood pressure, with their respective units, mean, and standard deviation (SD). We present categorical variables as frequency counts for gender, family history of diabetes, hypertension, cardiovascular disease, stroke, and diabetic status, which provide an overview of the key characteristics of the dataset.

**Table 2:** Summary statistics of continuous and categorical variables.

Continuous Variable	Mean ± SD	Unit	Categorical Variable	Category	Count
Age	45.75 ± 13.42	years	Gender	Female	3752
Pulse Rate	76.63 ± 12.23	bpm		Male	1536
Systolic BP	134.00 ± 22.23	mmHg	Family History of Diabetes	No	5119
				Yes	169
Diastolic BP	82.23 ± 12.48	mmHg	Hypertensive	No	4701
				Yes	587
Glucose	7.56 ± 2.94	mmol/L	Family History of Hypertension	No	5110
				Yes	178
Height	1.55 ± 0.08	m	Cardiovascular Disease	No	5228
				Yes	60
Weight	53.64 ± 10.08	kg	Stroke	No	5268
				Yes	20
BMI	22.47 ± 8.82	kg/m²	Diabetic Status	No	4946
				Yes	34

## 4 Experimental Design, Materials and Methods

This section outlines the comprehensive strategy and methodology employed for data collection and validation. The study involved 5,288 participants, carefully selected to represent a diverse Bangladeshi population across various ages, sexes, socioeconomic statuses, and geographic locations.

### 4.1 Limitations of existing diabetes data sets

Datasets like PIMA often lack demographic diversity among participants, leading to the overrepresentation of some groups and the exclusion of others. Additionally, the PIMA dataset exclusively focuses on Pima Indian women and lacks representation from males or other ethnic backgrounds. Similarly, datasets from different institutions like Ranchi University and Luzhou Hospital exhibit imbalances in gender distribution and lack comprehensive documentation on vital health parameters. These limitations restrict the generalizability and applicability of these datasets in broader research contexts.

### 4.2 Experimental design for data collection

**Fig. 2:**
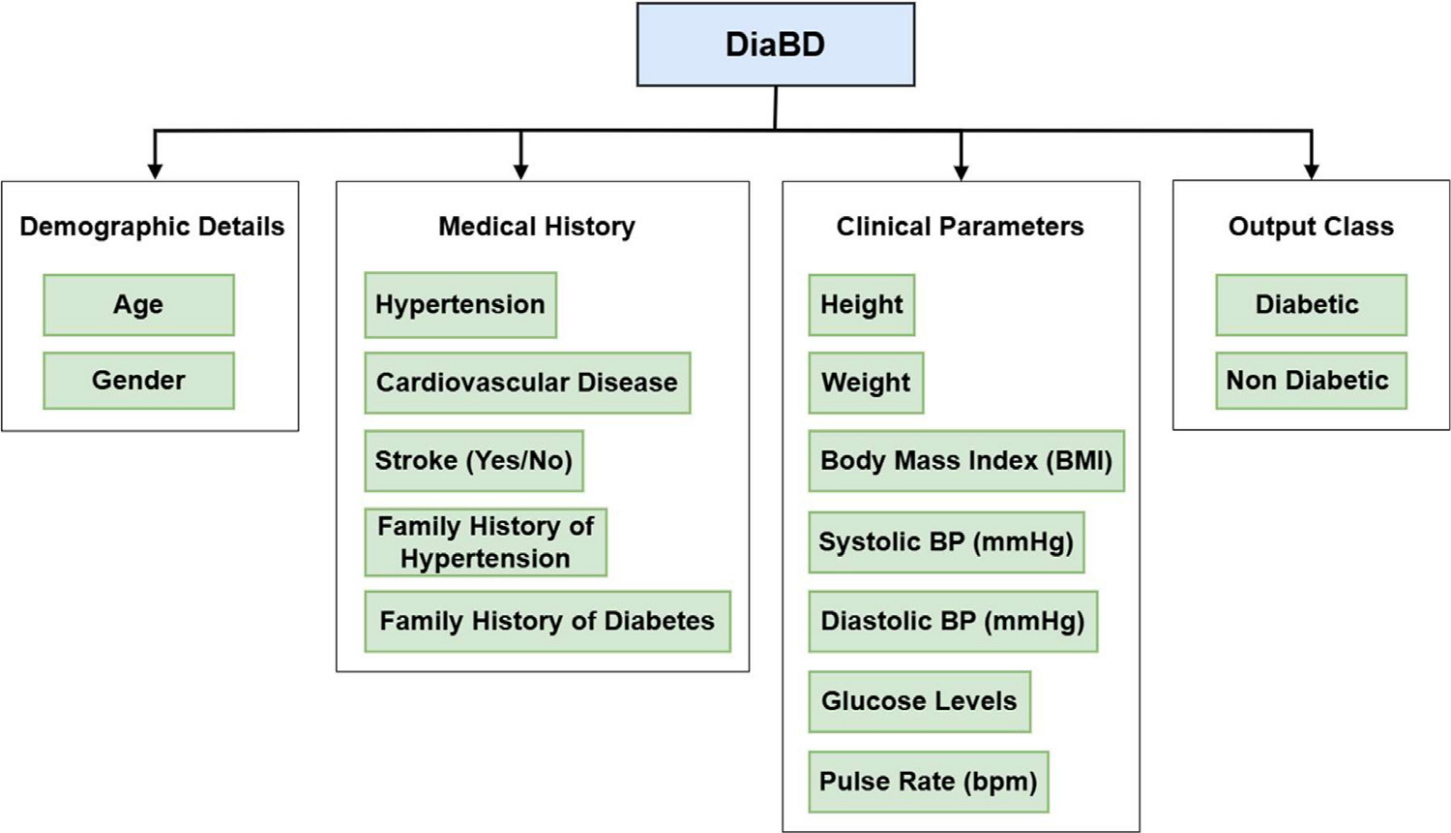
Overview of the dataset design with all relevant attributes.

Data encompasses clinical parameters, including systolic and diastolic blood pressures, BMI, glucose levels, and crucial demographic and lifestyle information such as family medical history and existing health conditions like hypertension and stroke. This comprehensive way of collecting data makes the dataset more helpful in creating targeted health interventions and strong prediction models for diabetes and the other conditions often found with it. The data collected also supports the examination of health trends and the development of personalized healthcare strategies, significantly advancing the field of medical research in Bangladesh and potentially other comparable regions. A dataset preparation flowchart is illustrated in Fig. 2.

### 4.3 Data collection methodology

The data in this paper were collected from a study conducted in different regions of Bangladesh, where community health workers (CHWs) used an innovative health kit and an Artificial Intelligence(AI)-based mobile app to provide weekly health education, screenings, risk assessments, and digital referrals. This study uses a repeated cross-sectional design to evaluate the impact of a digital health intervention on health outcomes in rural Bangladesh. Data collection occurred between April 10, 2021, and May 27, 2024, as a joint initiative by CMED Health and PKSF. Data was collected from 5,288 participants across 63 Unions in urban, semi-urban, and rural areas using random selection. CHWs obtained informed written consent from all participants and provided details about the study’s nature and risks.

CHWs visited each household once a week to collect their health vitals and input them into their digital profile through the ”Enriched Sastho” application. For this dataset, we selected participants aged 21-80 years, which is the most crucial age group for Type 2 diabetes (T2D) in Bangladesh [14]. According to a recent study [15], the prevalence of T2D was 10.8% in urban areas and 7.4% in rural areas, with 31.4% of the urban population and 27% of the rural population affected by pre-diabetes. Based on these percentages, we distributed participants in urban, semi-urban, and rural areas according to the population distribution to maintain demographic balance. Participants were randomly selected within this age group. The selected areas also had a higher concentration of migrations of populations from various regions across the country.

This study used a triangulation of quantitative, qualitative, and participant observation methods, following Action Research principles. Initially, it was conducted as a pilot project on digital health services in a union that consists of 21 villages of the Dhamrai sub-district, Dhaka, Bangladesh. CHWs were trained according to WHO guidelines for non-communicable diseases and received three months of training before data collection with a government-approved training module. Inter-rater reliability was ensured through this standardized training, a pilot test under the supervision of three groups of physicians to confirm consistency in data collection and recording across different areas. To mitigate selection bias, participants were randomly selected in the age range of 21 to 80 years from defined areas. We excluded participants who were not Bangladeshi citizens or did not meet the age criteria. This process ensured a representative sample of the Bangladeshi population.

Data were collected using a structured questionnaire (Table 1), with closed-ended questions for categorical variables and quantitative measures (e.g., blood pressure, glucose levels) utilizing validated instruments approved by CE/FDA included in the CMED health kit. The CMED Health Kit contains tools to measure height (Height Scale), weight (Weight Scale), blood pressure (Sphygmomanometer), blood sugar (Glucometer), blood oxygen saturation (Pulse Oximeter), and temperature (Thermometer). Additionally, it tracks BMI, pulse rate, and uses the MUAC tape to assess nutritional status.

The pilot project evaluated the validity of the questionnaire, translated it into Bangla, and verified it through back-translation. Community health workers visit participants weekly and collect data on health vitals, including blood pressure, glucose levels, and BMI, during every session. We select the most frequent and stable values from the collected data for analysis. For glucose levels, we test the participant’s glucose level randomly during each session. The questionnaire was filled out by trained CHWs, who asked the participants and recorded their responses. After data collection, three teams of physicians reviewed the data to ensure it met WHO standards, confirming its accuracy and reliability for analysis.

### 4.4 Data preprocessing

We performed error correction and data cleaning to remove inaccuracies and noise from the dataset. Initially, duplicate records identified and removed from an initial dataset of 5,373, resulting in 5,301 unique records. Subsequently, we excluded patient cases with missing data for any attributes to ensure the integrity and consistency of the dataset, which reduced the dataset to 5,288 records. Despite the varied data collection settings, each CHW followed the same protocol, measurement techniques, and tools to maintain data consistency. We standardized biometric measurements, including blood pressure, glucose levels, and BMI, and categorical data, such as gender labels and hypertension status, to maintain uniformity and consistency across the dataset. To maintain the raw form of the dataset, we allowed flexibility for applying normalization techniques based on specific analytical needs. We used consistent measurement techniques and tools across all settings during data collection to achieve reliable data.

### 4.5 Technical validation

A collaborative validation protocol ensured the accuracy and reliability of our diabetes-related dataset through a two-stage process. This validation process is designed to meet clinical standards and ensure clinical validity. The protocol included three independent groups of physicians from diverse medical backgrounds. If two teams agreed on a diabetic or non-diabetic classification, they finalized that without further review. In cases of disagreement, they escalated the flagged entries to a third group of physicians, who impartially reviewed the discrepancies and made the final, clinically valid recommendations. They corrected and confirmed errors and retained valid entries that were initially flagged as errors. This structured, consensus-driven validation framework bolstered the robustness and clinical reliability of the dataset.

**Fig. 3:**
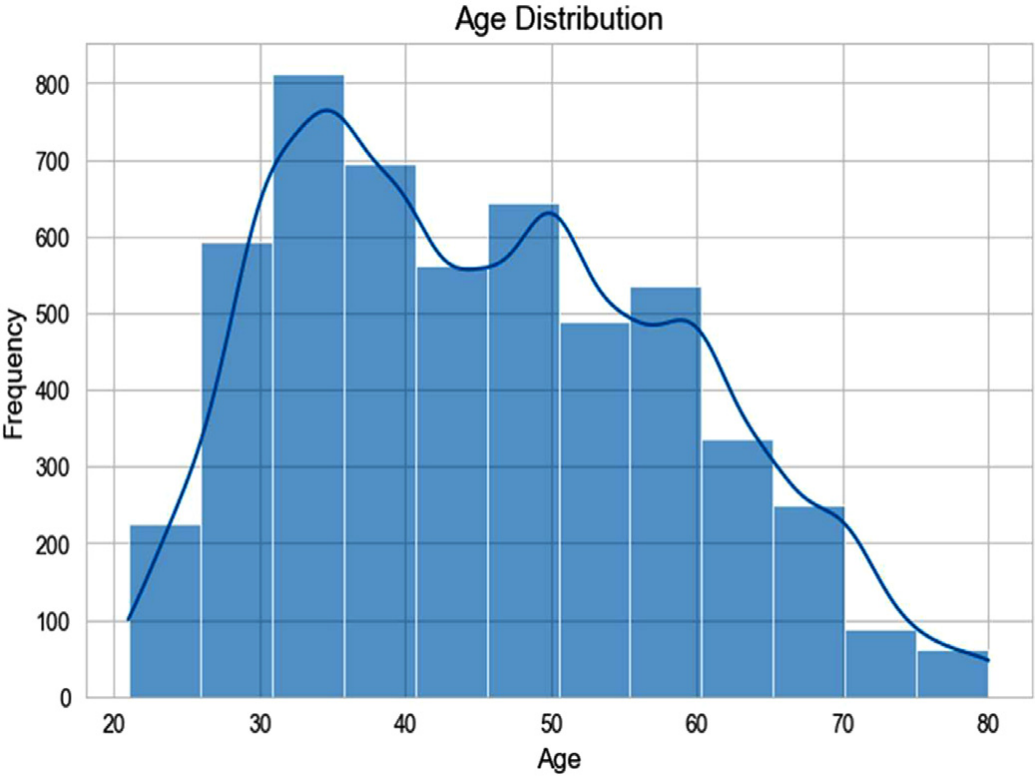
Age distribution in the dataset.

The dataset includes 70.95% female and 29.05% male participants. Regarding age distribution, the percentages for the age groups 21–29 years, 30–39 years, 40–49 years, 50–59 years, and over 60 years are 7.31%, 28.93%, 21.70%, 21.56%, and 20.16%, respectively. In terms of health conditions, 11.10% of patients are hypertensive, while 88.90% are not. Additionally, the dataset includes 0.38% of patients with a history of stroke and 1.13% with cardiovascular disease. Regarding medical history, 3.20% of patients report having a close relative with type 2 diabetes, and 3.76% have one or both parents diagnosed with hypertension. This dataset provides a comprehensive basis for diabetes and related health condition analysis. Fig. 3 illustrates the distribution of participants across different age groups. The most significant proportion is observed in the 30–39 years range. Followed by 40–49 years and 50–59 years, with smaller proportions in the 21–29 years and 60+ years groups.

**Fig. 4:**
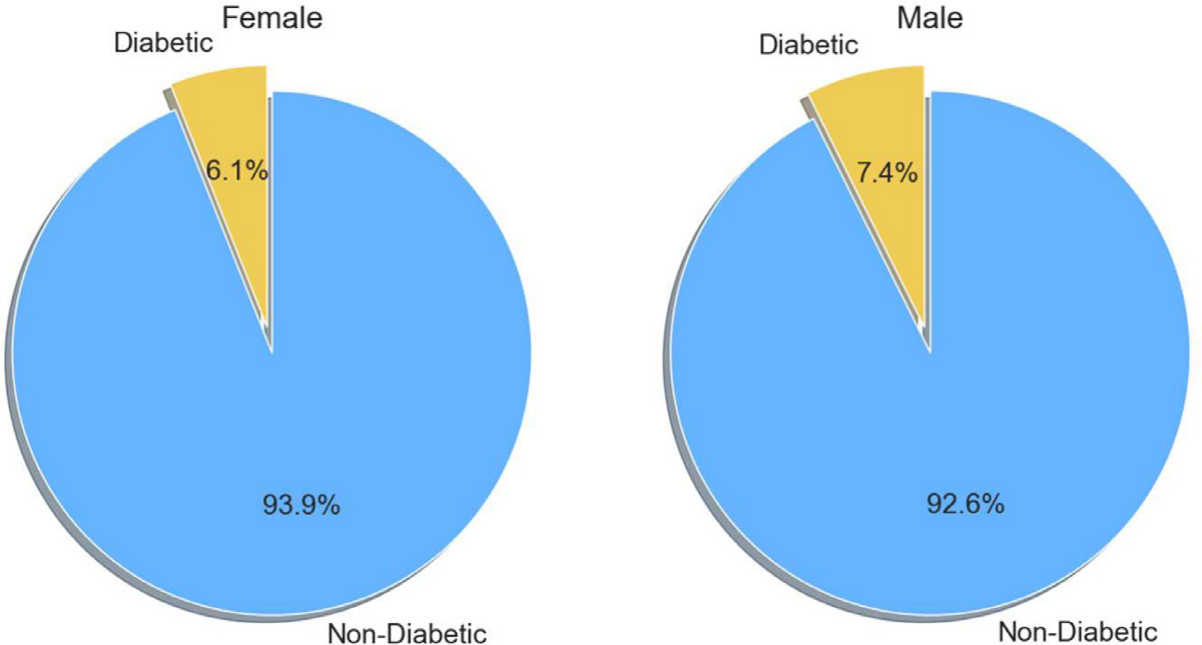
Percentage of diabetic and non-diabetic patients in the dataset.

There were 342 instances of diabetic patients among 5,288 cases. Among 342 samples, 228 (66.67%) were female with Diabetes ‘Yes’ class samples, and 114 (33.33%) were male with Diabetes ‘Yes’ class samples. The data also reveals a gender-based variation in diabetes prevalence. Among female participants, 6.1% are diagnosed with diabetes, while 93.9% are non-diabetic. In contrast, 7.4% of male participants have diabetes, whereas 92.6% do not. This data indicates a slightly higher frequency of diabetes among males than females within the dataset. Understanding such gender-based differences can provide valuable insights for targeted prevention strategies and personalized healthcare interventions, as shown in Fig. 4. Among 4946 non-diabetic patients, 1422 were male, and 3524 were female, with the number of females being 1.013 times higher than males.

**Fig. 5:**
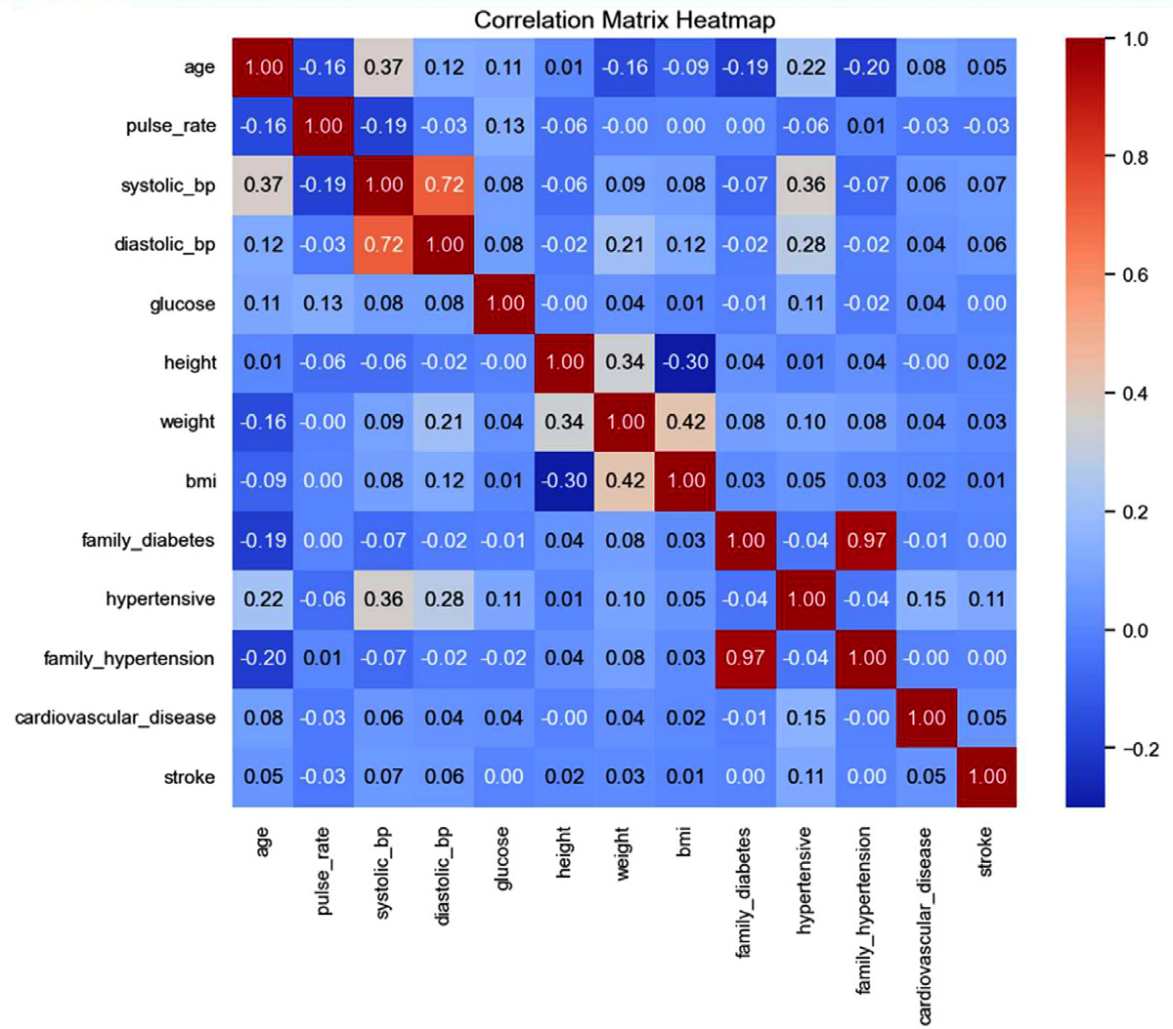
Correlation matrix of clinical and medical parameters.

A correlation matrix heatmap presented in Fig. 5, illustrates the relationships between clinical and medical parameters within the dataset. The color-coded matrix represents Pearson correlation coefficients, where red indicates a strong positive correlation, blue signifies a negative correlation, and white represents a lack of significant correlation. Systolic and diastolic blood pressure exhibit a strong positive correlation (0.72), highlighting their interdependence. BMI shows a moderate correlation with weight (0.42) and a slight negative correlation with height (-0.30), aligning with BMI calculation principles. A strong association (0.97) exists between family hypertension and hypertension, emphasizing genetic predisposition. Family diabetes has a moderate correlation with diabetes, reinforcing hereditary influences. Hypertension and CVD display a mild correlation (0.15), reflecting their clinical relationship, while CVD and stroke share a weaker correlation (0.10). These findings offer valuable insights into disease risk factors, aiding the development of predictive models for diabetes and related conditions.

## Limitations

This study offers valuable insights into diabetic studies, yet it is essential to acknowledge certain limitations. The dataset exhibits some imbalances; for instance, there are more participants without diabetes than with diabetes. Furthermore, there is a gender imbalance, with female participants almost twice as many as male participants. These imbalances will introduce statistical bias and may affect the generalizability and accuracy of the findings. The data was curated thoroughly, but there are biases and a lack of representation, which are critical factors that will affect the overall reliability of the study.

## Ethics Statement

The data collection and validation process adhered to ethical standards, with protocols approved by the ethical review board of United International University (UIU) (Reference No: IREB/2023/009). Robust security measures were implemented to protect all personal data and prevent unauthorized access. Informed written consent was obtained from all participants, who were fully informed about the nature of the study, potential risks, and their rights to withdraw at any time. They voluntarily agreed to participate after having all aspects of the study explained to them. To protect participant rights and dignity, we meticulously observed all ethical considerations throughout the study, including confidentiality, anonymity, and responsible sharing of data to maintain the highest ethical standards.

## CRediT Author Statement

**Tabia Tanzin Prama:** Conceptualization, Methodology, Visualization, Investigation, Data Curation, Data Validation, Formal Analysis, Writing—original draft, review, and editing. **Md. Jobayer Rahman:** Conceptualization, Methodology, Visualization, Investigation, Data Validation. Writing—original draft, review, and editing. **Marzia Zaman:** Data curation, data validation, Writing—review, and editing. **Farhana Sarkar:** Validation, Supervision, Methodology, Writing—review, and editing. **Khondaker A. Mamun:** Supervision, Conceptualization, Methodology, Visualization, Investigation, Data Curation, Data Validation, Formal Analysis, Writing—original draft, review, and editing.

## Data Availability

Mendeley DataDiaBD: A Diabetes Dataset for Enhanced Risk Analysis and Research in Bangladesh (Original data).
